# Supplementary material for: Role of Lung Ultrasound in Predicting Clinical Severity and Fatality in COVID-19 Pneumonia
Source: J Pers Med. 2021 Jul 30;11(8):757. doi: 10.3390/jpm11080757 (PMC8399683; doi:10.3390/jpm11080757)
Supplement: Supplementary file 1 [file jpm-11-00757-s001.zip › Supplementary file 2 - Mean infiltration scores by regions of the lungs (1).pdf]

Table S1 – Mean infiltration scores by regions of the lungs

	Mean (SD)	Minimum maximum	Median (IQR)
Region 1	2.26 (1.04)	0 – 3	3 (2 – 3)
Region 2	2.06 (1.00)	0 – 3	2 (2 – 3)
Region 3	1.36 (1.02)	0 – 3	1 (0.5 – 2)
Region 4	2.08 (1.10)	0 – 3	2 (2 – 3)
Region 5	1.89 (1.08)	0 – 3	2 (1 – 3)
Region 6	1.12 (1.07)	0 – 3	1 (0 – 2)
Region 7	1.94 (1.03)	0 – 3	2 (1 – 3)
Region 8	1.92 (1.02)	0 – 3	2 (1 – 3)
Region 9	2.00 (0.97)	0 – 3	2 (1 – 3)
Region 10	1.98 (1.00)	0 – 3	2 (1 – 3)
Region 11	1.35 (1.16)	0 – 3	2 (0 – 2)
Region 12	1.79 (1.14)	0 – 3	2 (1 – 3)
Region 13	1.52 (1.17)	0 – 3	2 (0 – 3)
Region 14	1.66 (1.27)	0 – 3	2 (0 – 3)
Basal	1.85 (0.70)	0 – 3	2 (1.5 – 2.3)
Middle	1.96 (0.79)	0 – 3	2 (1.5 – 2.5)
Upper	1.48 (0.80)	0 – 3	1.5 (1 – 2.2)*
Right	1.81 (0.68)	0 – 3	1.9 (1.4 – 2.3)
Left	1.75 (0.72)	0 – 3	1.9 (1.4 – 2.3)

*Friedmann test: F = 26.84, DF1 = 2, DF2 = 260, P < 0.001; multiple comparisons detected difference (P < 0.05) between upper regions and all other.
Acronyms: IQR – interquartile range; SD – standard deviation.

Table S2 – Ultrasonic according to highest respiratory support used

	Mean ± SD or Median (IQR) according to highest respiratory support modality used					P
	0L (n = 14)	1–10L (n = 69)	11–16L or Venturi (n = 20)	HFNC (n = 14)	MV (n = 14)	
LUS score	10 (3–21)	24 (19–26)	32 (27–35)	35 (32–38)	36 (31–38)	<0.0001
Age (years)	61.3 ± 16.0	63.8 ± 11.8	64.6 ± 10.4	64.6 ± 9.2	72.2 ± 8.7	0.115
Infiltration						
Upper region (0–18)	1.5 (0.0–3.0)	5.0 (4.0–7.0)	8.0 (6.0– 10.0)	9.5 (8.0–11.0)	8.5 (6.0–9.0)	<0.0001*
Middle region (0–6)	3.0 (0.0–7.0)	7.0 (5.0–9.0)	10.0 (9.5– 10.5)	11.0 (10.0–12.0)	11.5 (11.0–12.0)	< 0.0001†
Basal region (0–18)	4.5 (3.0–11.0)	11.0 (8.0–12.0)	13.0 (11.5–16.0)	14.5 (13.0–17.0)	15.5 (12.0–17.0)	<0.0001*

* Pairwise comparison of subgroups detected significant differences in: 0L vs. 1-10L, 0L vs. 10-16L, 0L vs. HFNC, 0L vs. MV, 1 – 10L vs. 10 – 16L, 1 – 10L vs. HFNC, and 1 – 10L vs. MV.
† Pairwise comparison of subgroups detected significant differences in : 0L vs. 1-10L, 0L vs. 10-16L, 0L vs. HFNC, 0L vs. MV, 1 – 10L vs. 10 – 16L, 1 – 10L vs. HFNC, 1 – 10L vs. MV and 11 – 16L vs. MV. Acronyms: HFNC – high flow nasal cannula; IQR – interquartile range; LUS score– lung ultrasound score; MV – mechanical ventilation.
