# Supplementary material for: Role of Lung Ultrasound in Predicting Clinical Severity and Fatality in COVID-19 Pneumonia
Source: J Pers Med. 2021 Jul 30;11(8):757. doi: 10.3390/jpm11080757 (PMC8399683; doi:10.3390/jpm11080757)
Supplement: Supplementary file 1 [file jpm-11-00757-s001.zip › Supplementary file 3 - Logistic regression according to highest respiratory support (mechanical ventilation) (1).pdf]

Table S1 – Univariate logistic regression according to highest respiratory support (0 – 16L vs. HFNC)

	ß	Wald	P	OR	95% CI
Age	0.007	0.09	0.77	1.01	0.96 – 1.06
LUS score	0.35	17.9	<0.001	1.42	1.21 – 1.68
Day of the illness	0.02	1.99	0.16	1.02	0.99 – 1.05
CRP (mg/L)	0.003	1.07	0.30	1.003	0.99 – 1.008
Leukocyte count (x 10^9/L)	0.08	1.68	0.20	1.09	0.96 – 1.23
Neutrophils (%)	0.06	2.5	0.11	1.06	0.98 – 1.15
Lymphocytes (%)	-0.06	1.88	0.17	0.94	0.86 – 1.03
D–dimer (μg/L)	0.004	0.002	0.96	1.004	0.85 – 1.19
LDH (U/L)	0.002	1.32	0.25	1.002	0.99 – 1.01
spO2 (%)	-0.03	1.29	0.26	0.97	0.93 – 1.02
pO2 (kPa)	-0.41	2.38	0.12	0.66	0.39 – 1.12
Comorbidity					
Arterial hypertension	0.64	0.86	0.35	1.89	0.49 – 7.21
Diabetes	0.17	0.07	0.79	1.18	0.34 – 4.10
Cardiovascular	0.51	0.62	0.43	1.66	0.47 – 5.84
Malignancy	0.83	1.29	0.26	2.28	0.55 – 9.45
Infiltration					
Upper region (0 – 12)	0.42	6.63	**0.01**	1.53	1.11 – 2.10
Middle region (0 – 12)	0.61	4.93	**0.03**	1.84	1.07 – 3.15
Basal region (0 – 18)	0.19	2.42	0.12	1.21	0.95 – 1.56
Anterior (0 – 12)	0.31	4.57	0.03	1.36	1.03 – 1.79
Lateral (0 – 12)	0.56	6.20	**0.01**	1.75	1.13 – 2.72
Posterior (0 – 18)	0.32	5.80	**0.02**	1.38	1.06 – 1.78
Left (0 – 21)	0.26	3.92	0.05	1.30	1.003 – 1.69
Right (0 – 21)	0.44	9.55	**0.002**	1.56	1.18 – 2.06

Acronyms: CI – confidence interval, HFNC high flow nasal cannula, LUS score – lung ultrasound score, OR – odds ratio.

Table S2 – Multivariate regression analysis according to highest respiratory support (0 – 16L vs. HFNC)

	ß	Wald	P	OR	95% CI
LUS score	0.353	17.918	<0.001	1.42	1.21 – 1.68
Constant	-12.414	20.964	<0.001		

Two independent predictors (Stepwise method) gave a unique statistically significant contribution to the model and these are LUS and age (Hosmer - Lemeshow test, P = 0.868). The model as a whole is statistically significant ($\chi 2$ = 336.81, P < 0.001), and explains between 27.0% (according to Cox & Snell) and 52.0% (according to Negelkerke) the variance of the negative outcome (necessity of NFHC oxygen administration), and accurately classifies 90.6% of cases.

Table S3 – Univariate logistic regression according to highest respiratory support (mechanical ventilation)

	ß	Wald	P	OR	95% CI
Age	0.076	6.309	**0.012**	1.08	1.02 - 1.15
LUS score	0.220	14.348	**< 0.001**	1.25	1.11 - 1.40
Day of the illness	-0.133	3.869	**0.049**	0.88	0.77 - 1.00
CRP (mg/L)	0.001	0.031	0.861	1.00	0.99 - 1.01
Leukocyte count (x 10^9/L)	-0.031	0.161	0.688	0.97	0.83 - 1.13
Neutrophils (%)	-0.001	0.001	0.978	1.00	0.94 - 1.06
Lymphocytes (%)	0.003	0.005	0.943	1.00	0.93 - 1.08
D–dimer (µg/L)	0.025	0.096	0.756	1.03	0.88 - 1.20
LDH (U/L)	0.002	2.058	0.152	1.00	1.00 - 1.01
Troponin (ng/L)	0.005	0.081	0.776	1.00	0.97 - 1.04
spO2 (%)	-0.026	0.626	0.429	0.97	0.91 - 1.04
pO2 (kPa)	-0.317	1.135	0.287	0.73	0.41 - 1.31
Comorbidity					
Arterial hypertension	-0.262	0.188	0.664	0.77	0.24 - 2.51
Diabetes	-0.139	0.041	0.840	0.87	0.22 - 3.37
Cardiovascular	0.885	2.073	0.150	2.42	0.73 - 8.07
Malignancy	-0.489	0.206	0.650	0.61	0.07 - 5.08
Infiltration					
Upper region (0 – 12)	0.275	6.799	**0.009**	1.32	1.07 - 1.62
Middle region (0 – 12)	1.059	12.554	**< 0.001**	2.88	1.61 - 5.18
Basal region (0 – 18)	0.354	10.550	**0.001**	1.42	1.15 - 1.76
Anterior (0 – 12)	0.244	6.438	**0.011**	1.28	1.06 - 1.54
Lateral (0 – 12)	0.575	10.203	**0.001**	1.78	1.25 - 2.53
Posterior (0 – 18)	0.429	11.840	**0.001**	1.54	1.20 - 1.96
Left (0 – 21)	0.321	12.081	**0.001**	1.38	1.15 - 1.65
Right (0 – 21)	0.378	12.227	**0.001**	1.46	1.18 - 1.80

Acronyms: LUS – lung ultrasound score; OR – odds ratio;

Table S4 – Multivariate regression analysis according to highest respiratory support (mechanical ventilation)

	ß	Wald	P	OR	95% CI
Age	0.081	5.051	0.025	1.08	1.01 – 1.16
LUS score	0.215	12.617	<0.001	1.24	1.10 – 1.40
Constant	-14.044	17.192	<0.001		

Two independent predictors (Stepwise method) gave a unique statistically significant contribution to the model and these are LUS and age (Hosmer - Lemeshow test, P = 0.89). The model as a whole is statistically significant ($\chi2$ = 28.20, P < 0.001), and explains between 19.4% (according to Cox & Snell) and 39.3% (according to Negelkerke) the variance of the negative outcome, and accurately classifies 91.6% of cases.
