# Supplementary material for: Role of Lung Ultrasound in Predicting Clinical Severity and Fatality in COVID-19 Pneumonia
Source: J Pers Med. 2021 Jul 30;11(8):757. doi: 10.3390/jpm11080757 (PMC8399683; doi:10.3390/jpm11080757)

Figure S1 - Receiver operating curve analysis of sensitivity, specificity, and cut-off values for LUS score and age according to highest respiratory modality (mechanical ventilation) in comparison to D-dimer demonstrating the strength of lung ultrasound over blood analysis.

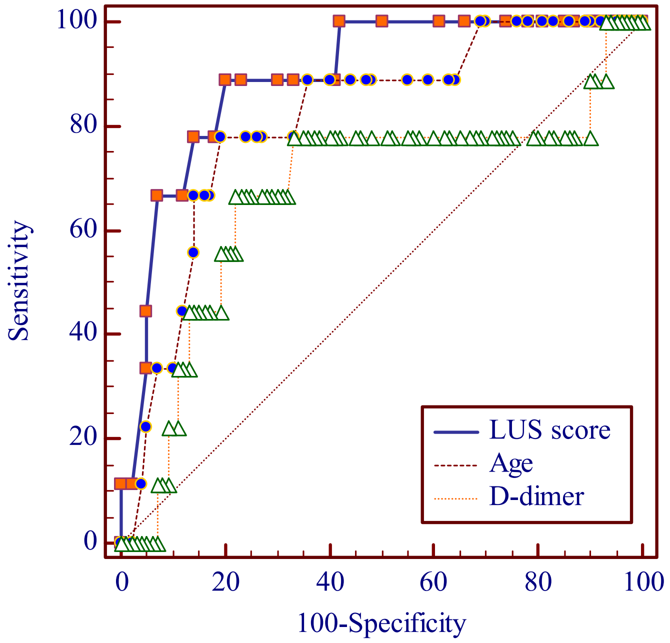

Figure S2 - Receiver operating curve analysis of sensitivity, specificity, and cut-off values for LUS score and age according to the probability of a negative outcome (death) in comparison to D-dimer demonstrating the strength of lung ultrasound over blood analysis.

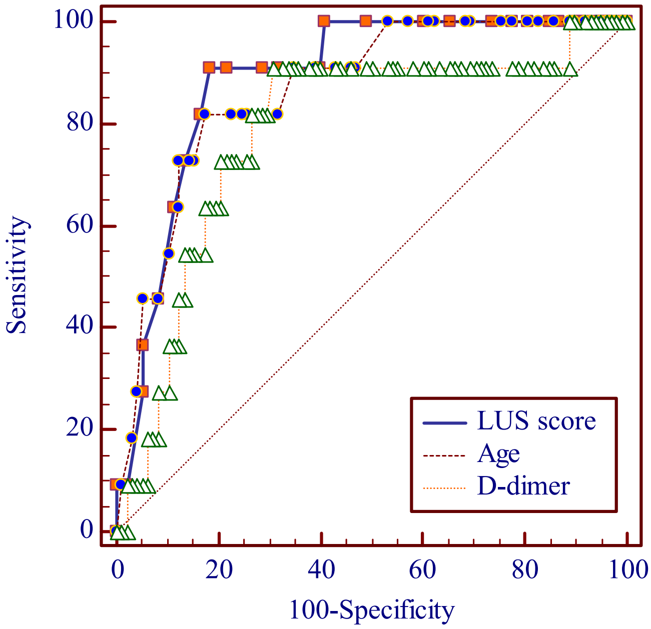

Supplement: Supplementary file 1 [file jpm-11-00757-s001.zip › Supplementary file 4 - ROC LUS over LAB.pdf]
