# Supplementary material for: Role of Lung Ultrasound in Predicting Clinical Severity and Fatality in COVID-19 Pneumonia
Source: J Pers Med. 2021 Jul 30;11(8):757. doi: 10.3390/jpm11080757 (PMC8399683; doi:10.3390/jpm11080757)
Supplement: Supplementary file 1 [file jpm-11-00757-s001.zip › Supplementary file 5 - Logistic regression according to mortality (1).pdf]

Table S1 – Univariate logistic regression according to mortality

	ß	Wald	P	OR	95% CI
Age	0.151	15.658	**<0.001**	**1.16**	1.08 – 1.25
LUS score	0.260	17.792	**<0.001**	**1.30**	1.15 – 1.46
Day of the illness	0.021	1.921	0.166	1.02	0.99 – 1.05
CRP (mg/L)	0.000	0.007	0.935	1.00	0.99 – 1.01
Leukocyte count (x 10^9/L)	0.019	0.079	0.778	1.02	0.89 – 1.16
Neutrophils (%)	0.037	1.116	0.291	1.04	0.97 – 1.11
Lymphocytes (%)	–0.059	1.719	0.190	0.94	0.86 – 1.03
D–dimer (µg/L)	0.059	0.908	0.341	1.06	0.94 – 1.20
LDH (U/L)	0.003	3.681	0.055	1.00	1.00 – 1.01
Troponin (ng/L)	0.024	2.869	0.090	1.02	1.00 – 1.05
spO2 (%)	–0.035	1.366	0.243	0.97	0.91 – 1.02
pO2 (kPa)	–0.216	0.637	0.425	0.81	0.47 – 1.37
Comorbidity					
Arterial hypertension	0.758	1.262	0.261	2.13	0.57 – 8.00
Diabetes	0.075	0.015	0.904	1.08	0.32 – 3.65
Cardiovascular	1.632	8.170	**0.004**	**5.12**	1.67 – 5.67
Malignancy	1.235	3.455	**0.063**	**3.44**	0.93 – 2.64
Infiltration					
Upper region (0 – 12)	0.448	13.355	**<0.001**	**1.56**	1.23 – 1.99
Middle region (0 – 12)	0.821	12.957	**<0.001**	**2.27**	1.45 – 3.56
Basal region (0 – 18)	0.358	11.933	**0.001**	**1.43**	1.17 – 1.75
Anterior (0 – 12)	0.330	10.682	**0.001**	**1.39**	1.14 – 1.69
Lateral (0 – 12)	0.538	10.879	**0.001**	**1.71**	1.24 – 2.36
Posterior (0 – 18)	0.474	14.201	**<0.001**	**1.61**	1.26 – 2.06
Left (0 – 21)	0.406	16.437	**<0.001**	**1.50**	1.23 – 1.83
Right (0 – 21)	0.396	14.208	**<0.001**	**1.49**	1.21 – 1.83

Acronyms: LUS score – lung ultrasound score; OR – odds ratio;

Table S2 – Multivariate regression analysis according to mortality

	ß	Wald	P	OR	95% CI
Age	0.232	13.510	**<0.001**	**1.26**	1.14 – 1.43
LUS score	0.344	14.589	**<0.001**	**1.41**	1.18 – 1.68
Constant	-28.864	18.882	**<0.001**		

Two independent predictors (Stepwise method) gave a unique statistically significant contribution to the model and these are LUS and age (Hosmer - Lemeshow test, P = 0.41). The model as a whole is statistically significant ($\chi2 = 57.49$, $P < 0.001$), and explains between 35.5% (according to Cox & Snell) and 67.8% (according to Negelkerke) the variance of the negative outcome, and accurately classifies 95.4% of cases.
