# Supplementary material for: Role of Lung Ultrasound in Predicting Clinical Severity and Fatality in COVID-19 Pneumonia
Source: J Pers Med. 2021 Jul 30;11(8):757. doi: 10.3390/jpm11080757 (PMC8399683; doi:10.3390/jpm11080757)

Table S1 – Multivariate regression analysis according to highest respiratory support (mechanical ventilation) with all ultrasonic parameters included

	ß	Wald	P	OR	95% CI
Age	0.078	4.314	0.038	1.08	1.00 – 1.16
Infiltration in middle region	1.049	10.871	0.001	2.86	1.53 – 5.33
Constant	-17.892	15.299	<0.001		

Two independent predictors (Stepwise method) gave a unique statistically significant contribution to the model and these are age and infiltration in middle regions (Hosmer - Lemeshow test, P = 0.94). The model as a whole is statistically significant ($\chi2 = 34.72$, $P < 0.001$), and explains between 23.3% (according to Cox & Snell) and 47.2% (according to Negelkerke) the variance of the negative outcome, and accurately classifies 93.9% of cases.

Table S2 – Receiver operating characteristic curve parameters according to highest respiratory support (mechanical ventilation) with all ultrasonic parameters included

Parameters	AUC	95% CI	Sensitivity	Specificity	Cutoff	Youden index	*P*	*P* Age vs. Infiltration in middle region
Age	0.714	0.629 – 0.790	71.4	65.8	>68	0.38	0.001	0.013
Infiltration in middle region	0.891	0.824 – 0.938	78.6	85.5	>10	0.64	<0.001	

AUC = area under curve

Figure S1 – Receiver operating curve analysis for age and infiltration of the middle region according to highest respiratory modality (mechanical ventilation) with all ultrasonic parameters included

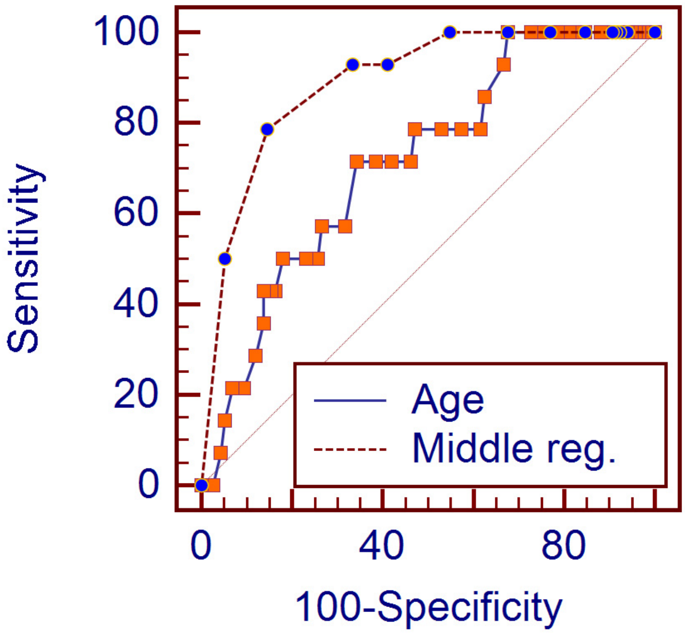

Supplement: Supplementary file 1 [file jpm-11-00757-s001.zip › Supplementary file 6 - Multivariate and ROC analysis when all ultrasonic parameters included.pdf]
